# Receptor-Ligand Interaction Mediates Targeting of Endothelial Colony Forming Cell-derived Exosomes to the Kidney after Ischemic Injury

**DOI:** 10.1038/s41598-018-34557-7

**Published:** 2018-11-05

**Authors:** Jose L. Viñas, Matthew Spence, Alex Gutsol, William Knoll, Dylan Burger, Joseph Zimpelmann, David S. Allan, Kevin D. Burns

**Affiliations:** 1 Division of Nephrology, Dept. of Medicine, Kidney Research Centre, Ottawa Hospital Research Institute, University of Ottawa, Ottawa, Ontario Canada; 2 Division of Hematology, Dept. of Medicine, Ottawa Hospital Research Institute, University of Ottawa, Ottawa, Ontario Canada

## Abstract

Endothelial colony forming cell (ECFC)-derived exosomes protect mice against ischemic kidney injury, via transfer of microRNA-(miR)-486-5p. Mechanisms mediating exosome recruitment to tissues are unclear. We hypothesized that ECFC exosomes target ischemic kidneys, involving interaction between exosomal CXC chemokine receptor type 4 (CXCR4) and stromal cell-derived factor (SDF)-1α. Ischemia-reperfusion was induced in mice by bilateral renal vascular clamp, with intravenous infusion of exosomes at reperfusion. Optical imaging determined exosome biodistribution, and miR-486-5p was measured by real-time PCR. Human umbilical vein endothelial cells (HUVECs) were cultured to study the CXCR4/SDF-1α interaction. Targeting of administered exosomes to ischemic kidneys was detected 30 min and 4 hrs after reperfusion. Exosomes increased miR-486-5p levels only in kidneys, within proximal tubules, glomeruli, and endothelial cells. Uptake of fluorescently-labeled exosomes into HUVECs, and exosomal transfer of miR-486-5p were enhanced by hypoxia, effects blocked by neutralizing antibody to SDF-1α or by the CXCR4 inhibitor plerixafor. Infusion of ECFC exosomes prevented ischemic kidney injury *in vivo*, an effect that was not observed when exosomes were pre-incubated with plerixafor. These data indicate that ECFC exosomes selectively target the kidneys after ischemic injury, with rapid cellular transfer of miR486-5p. Targeting of exosomes may involve interaction of CXCR4 with endothelial cell SDF-1α.

## Introduction

Acute kidney injury (AKI) is a clinical disorder characterized by rapid loss of kidney function occurring over hours to days, and is often due to ischemia/reperfusion injury, sepsis or nephrotoxins. AKI affects approximately 5% of hospitalized patients, is associated with high morbidity and mortality, and there are no established treatments that accelerate renal parenchymal repair in humans^1^. While proximal tubular cell damage is a characteristic feature of AKI, severe disturbances in renal microvascular blood flow and endothelial cell function also occur, leading to cell swelling, loss of glycocalyx, increased expression of adhesion molecules, and enhanced recruitment of leukocytes, which cause prolonged extension of the tubular injury^2^. Thus, in patients at risk of AKI, prevention of endothelial cell injury may represent a potential therapeutic strategy to preserve long-term kidney function.

Asahara *et al*. first described the isolation of “endothelial progenitor cells” (EPCs) from human peripheral blood in 1997, and showed that these cells express endothelial cell surface markers and promote neovascularization^3^. Subsequent animal studies revealed that administration of EPCs exerts protective effects in AKI^4–6^, hindlimb ischemia^7^, and ischemic retinopathy^8^. Homing of progenitor cells to sites of ischemia appears to play an important role in mediating the protective effects, and could involve cell surface B1 and B2 integrin expression, the G protein-coupled receptor C-X-C chemokine receptor type 4 (CXCR4), and interaction with chemokines such as stromal cell-derived factor (SDF)-1α^9–11^. Despite these homing mechanisms, engraftment of progenitor cells within injured tissues has not been consistently demonstrated, and the protective effects of EPCs have been attributed to paracrine mechanisms.

In this regard, extracellular vesicles have emerged as leading candidates mediating the protective effects of EPCs in models of ischemic tissue injury. One distinct class of extracellular vesicles is exosomes (diameter 40–100 nm), which are generated in the multivesicular body and then released from the cell^12^. Exosomes are packaged with cargo, including microRNA (miR), mRNA and proteins, and upon binding to other cells the exosomal contents may be transferred, thereby modulating cell function. Cantaluppi *et al*. showed that infusion of microvesicles of diameter 50–200 nm derived from human EPCs protected against kidney ischemic injury in rats^13^. In a mouse model of kidney ischemia, we demonstrated that intravenous infusion of exosomes derived from human cord blood endothelial colony forming cells (ECFCs) reduced kidney ischemic injury, associated with decreased histologic damage, apoptosis, and neutrophil infiltrate^14^. ECFC exosomes were found to be highly enriched in miR-486–5p, which targets *phosphatase and tensin homolog* (PTEN), thereby enhancing phosphorylation of Akt, and leading to inhibition of apoptosis^14^. Furthermore, infusion of ECFC exosomes at the time of reperfusion in mice with kidney ischemia was associated with an increase in kidney levels of miR-486-5p after 24 hrs, suggesting transfer of miR-486-5p to the injured kidney. Whether other organs experience an increase in miR-486-5p after exosome infusion is unclear.

In the present studies, we tested the hypothesis that infused ECFC exosomes target the kidneys in mice with ischemia/reperfusion injury, leading to selective transfer of miR-486-5p. We also studied the potential role of exosomal CXCR4 and SDF-1α in mediating selective targeting of exosomes in ischemic injury.

## Results

### Characterization of exosomes

**Figure 1:**
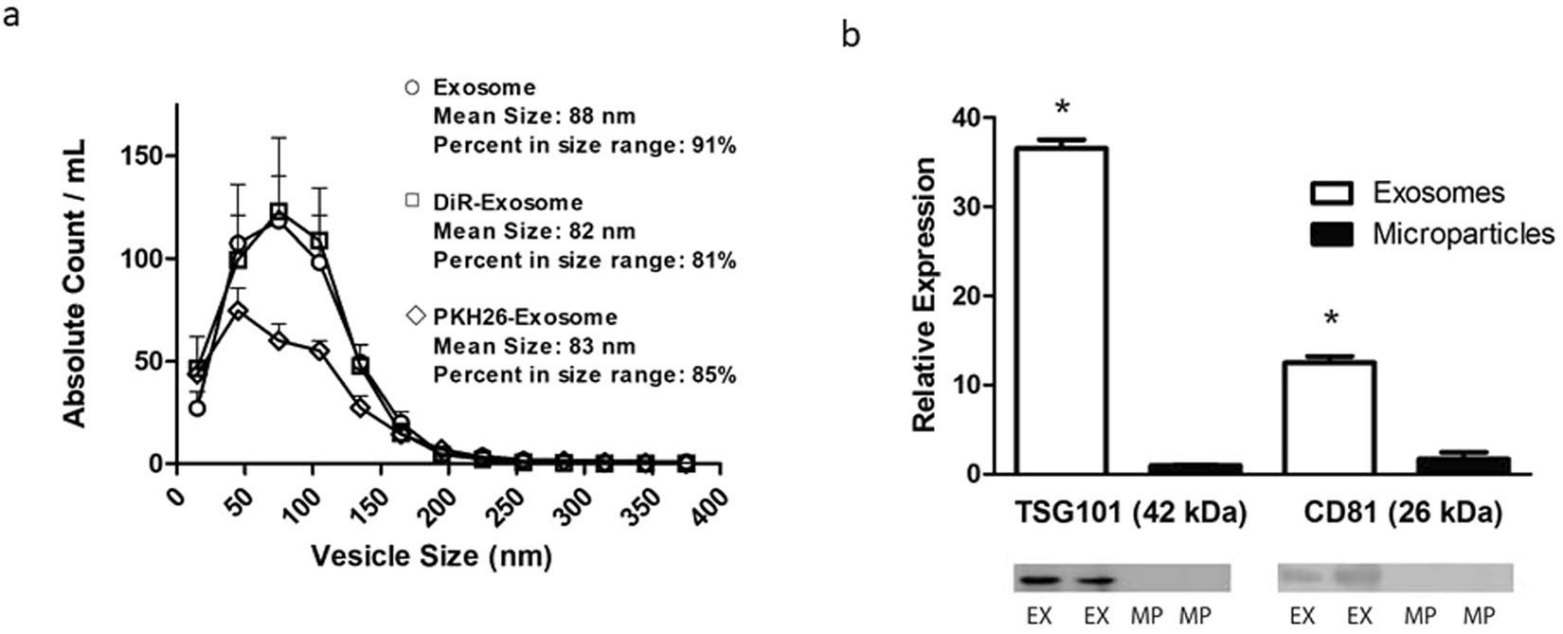
Characterization of human endothelial colony forming cell (ECFC)-derived exosomes (EX). (**a**) Graph depicts size distributions of unlabeled exosomes, as well as exosomes labeled with DiR or PKH26, isolated from ECFC conditioned medium, by nanoparticle tracking analysis. Data are mean ± SEM; n = 3. (**b**) Immunoblot analysis and graphic representation of exosomal markers TSG101 and CD81 in exosomes (EX) and larger extracellular vesicle (microparticles, MP) preparations. Data are mean ± SEM. *P < 0.001 vs microparticles, by unpaired Student t-test. Immunoblot image is representative of 3 independent experiments. Image of entire immunoblot is depicted in Supplementary Fig. 4a,b.

Exosomes were isolated from the conditioned media of ECFCs by serial centrifugation and characterized by nanoparticle tracking assay and immunoblots. As shown in Fig. 1, exosomes had a characteristic size distribution (mean diameter 88 nm, n = 3) and expressed Tumor Susceptibility Gene (TSG) 101 and CD81, which were absent in the larger diameter (100–1000 nm) extracellular vesicle population. Furthermore, the size distribution of exosomes was not affected by labeling with the lipophilic near-infrared dye 1,1′-dioctadecyltetramethyl indotricarbocyanine iodide (DiR) or the red fluorescent dye PKH26 (Fig. 1a). Zetaview images demonstrated characteristic Brownian motion of vesicles in the exosome fraction (Suppl S1, video).

### Biodistribution of ECFC exosomes after kidney ischemia/reperfusion

**Figure 2:**
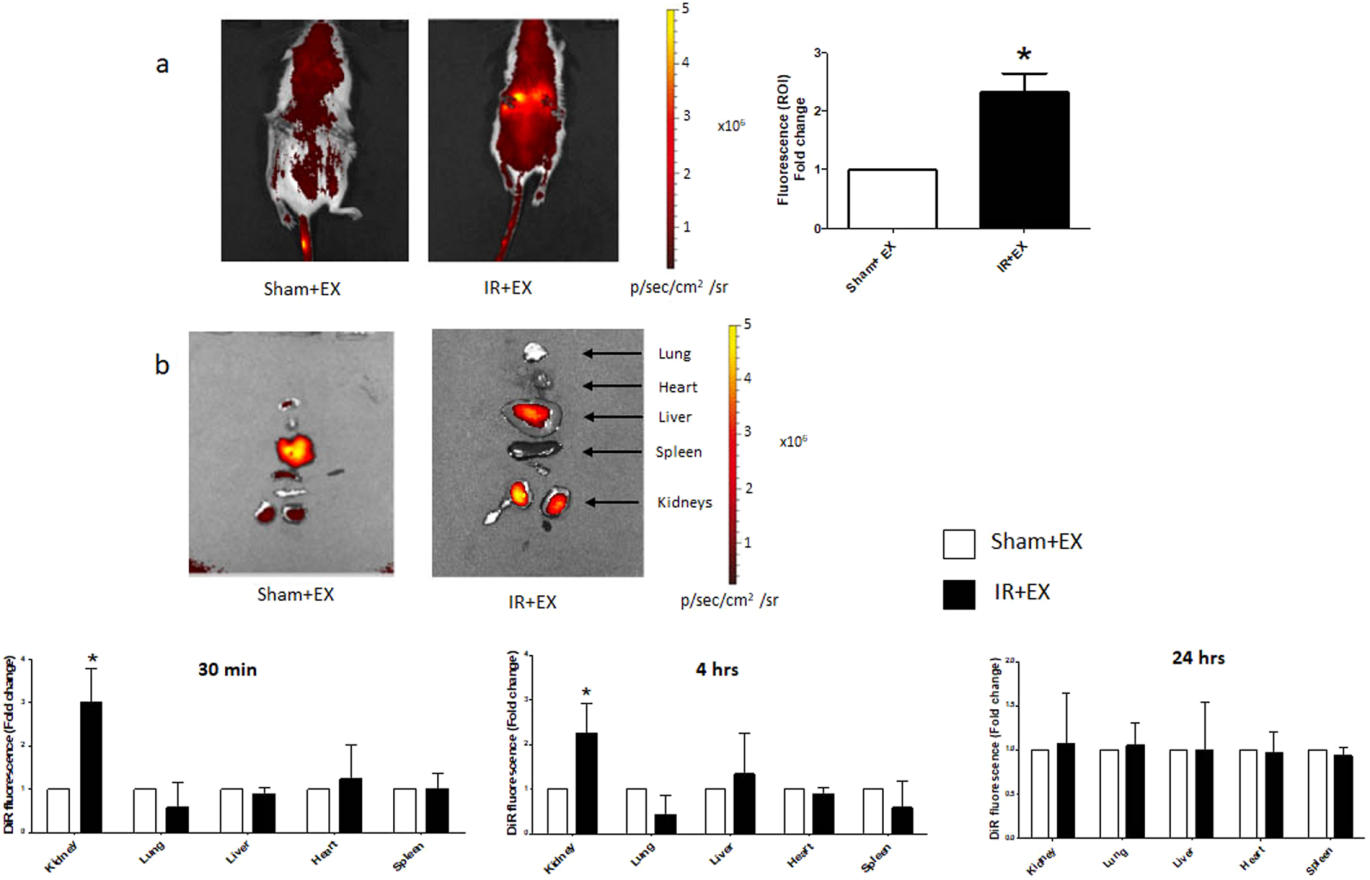
ECFC exosome biodistribution by optical imaging. Representative IVIS images in a live mouse (**a**) and in dissected organs (**b**) 30 min after injection of 20 μg DiR-labeled exosomes. Sham+EX (sham mice treated with exosomes), IR+EX (mice subjected to 30 min of kidney ischemia followed by infusion of exosomes). Graphs depict the fold-change in fluorescence between Sham+EX and IR+EX mice in the region of interest (ROI) (**a**) and in kidneys and other dissected organs at 30 min, 4 hrs and 24 hrs after DiR-exosome injection (**b**). *P < 0.01 vs Sham + EX by Student t-test **(a**) or one-way ANOVA (**b**) n = 4.

The biodistribution of infused DiR-labeled ECFC-derived exosomes was studied using the IVIS Spectrum in live mice and in isolated organs. In mice with ischemia/reperfusion kidney injury treated with DiR-labeled exosomes at the time of reperfusion, fluorescence was enhanced in the region around the kidneys at 30 min, compared to sham mice treated with DiR-labeled exosomes (Fig. 2a). Studies in isolated organs from mice infused with exosomes after kidney ischemia demonstrated a significant increase in kidney fluorescence 30 min and 4 hrs after reperfusion, but not at 24 hrs, compared to sham mice (Fig. 2b). Other organs did not demonstrate increases in DiR fluorescence after exosome infusion.

### Effect of exosomes on tissue levels of miR-486-5p after kidney ischemia/reperfusion

**Figure 3:**
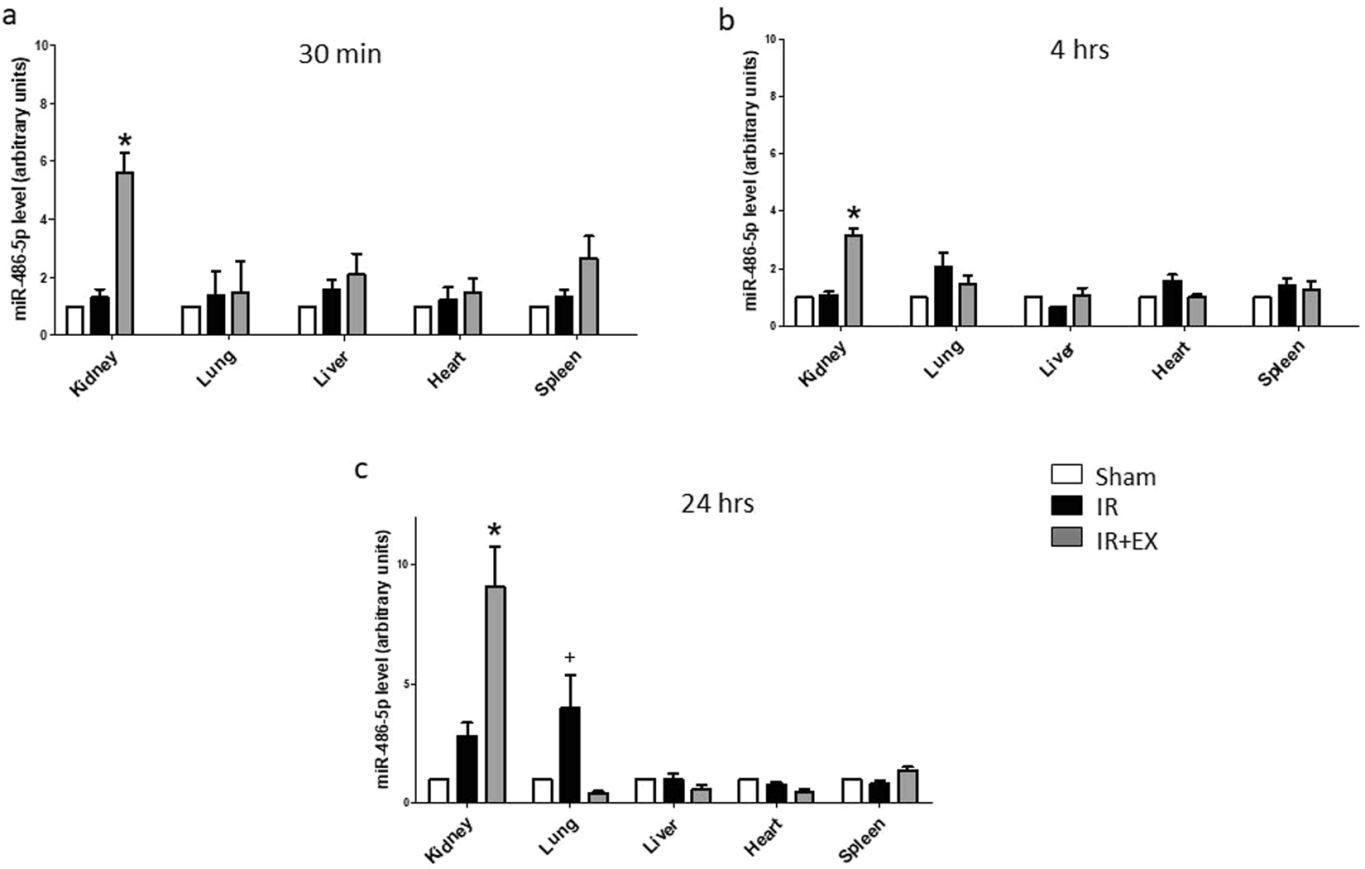
Exosomes selectively increase miR-486-5p levels in kidneys. Semi-quantitative analysis of miR-486-5p levels in organs of interest 30 min, 4 hrs and 24 hrs post-reperfusion. Sham (untreated mice), IR (mice with 30 min of bilateral kidney ischemia) and IR + EX (mice with 30 min of bilateral kidney ischemia followed by infusion of 20 μg of exosomes). *P < 0.01 vs Sham or IR, ^**+**^P < 0.05 vs Sham, by one-way ANOVA, n = 4.

To determine the effect of exosomes on tissue levels of miR-486-5p, mice were subjected to sham surgery or bilateral kidney ischemia for 30 min, and then infused with or without exosomes (20 µg i.v.) at the time of reperfusion. As shown in Fig. 3, ischemia/reperfusion alone did not alter miR-486-5p levels in any tissues, compared to sham mice, although a significant increase was found in lungs 24 hrs after reperfusion. Exosome infusion did not affect miR-486-5p levels in lung, heart, liver, or spleen at 30 min, 4 hrs or 24 hrs after reperfusion. By contrast, exosome infusion was associated with a significant increase in kidney levels of miR-486-5p at each time point.

**Figure 4:**
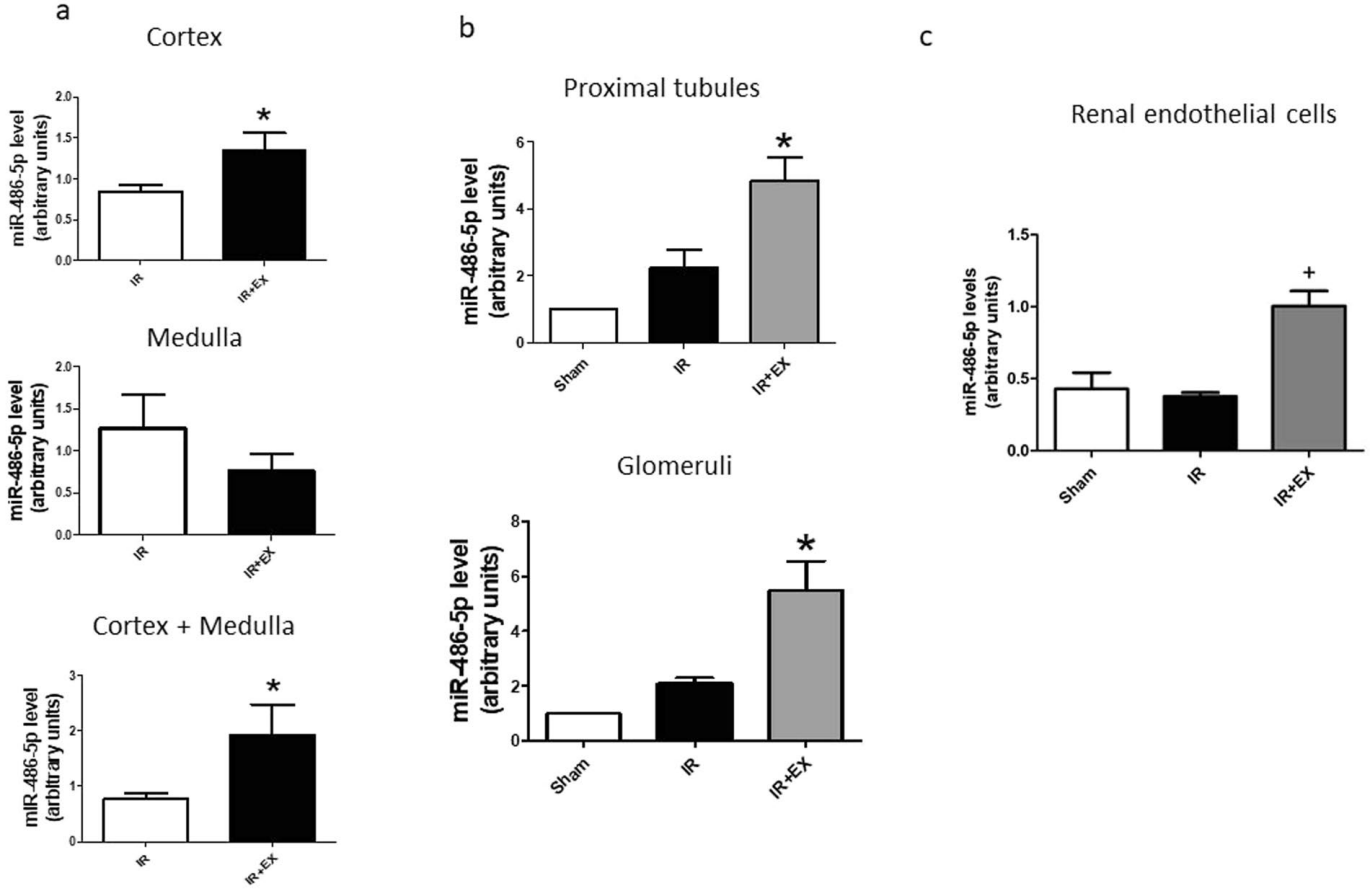
Exosomes increase miR-486-5p levels in kidney cortex, proximal tubules, glomeruli and kidney endothelial cells. Depicted are results for real-time PCR of miR-486-5p in renal cortex, medulla and whole kidney (**a**) dissected proximal tubules and glomeruli (**b**) and isolated kidney endothelial cells (**c**) Sham (untreated mice), IR (mice with 30 min of bilateral kidney ischemia) and IR+EX (mice with 30 min of bilateral kidney ischemia followed by infusion of 20 μg of exosomes). *P < 0.05 vs IR, ^**+**^P < 0.005 vs IR by one-way ANOVA, n = 4 for each panel.

Separate experiments were performed to determine the relative distribution of miR-486-5p in kidney cortex and medulla in mice treated with exosomes. Figure 4a shows that infusion of exosomes caused a significant increase in kidney cortical levels of miR-486-5p at 30 min after reperfusion, while levels in the medulla were not affected, compared to mice with ischemia/reperfusion alone.

Kidney cortices from mice subjected to sham surgery or kidney ischemic injury, with or without administration of exosomes, were subjected to collagenase digestion and microdissection of proximal tubular segments and glomeruli, 30 min after reperfusion. As shown in Fig. 4b, kidney ischemia-reperfusion alone led to non-significant increases in miR-486-5p levels in proximal tubules and glomeruli. However, infusion of ECFC exosomes caused significant increases in miR-486-5p levels in tubular segments and glomeruli, compared to ischemia/reperfusion alone (P < 0.05; n = 4).

To determine miR-486-5p levels in kidney endothelial cells, harvested kidneys were subjected to enzymatic digestion and endothelial cell sorting using CD31-labeled magnetic beads. Cells isolated and cultured after sorting demonstrated strongly positive immunofluorescent staining for CD31 (not shown). Figure 4c shows that miR-486-5p levels did not differ in isolated endothelial cells from sham-treated mice compared to mice with kidney ischemia/reperfusion alone. By contrast, infusion of exosomes caused a significant increase in kidney endothelial cell miR-486-5p level (P < 0.005; n = 4).

### Localization of exosomes in kidney tissue

**Figure 5:**
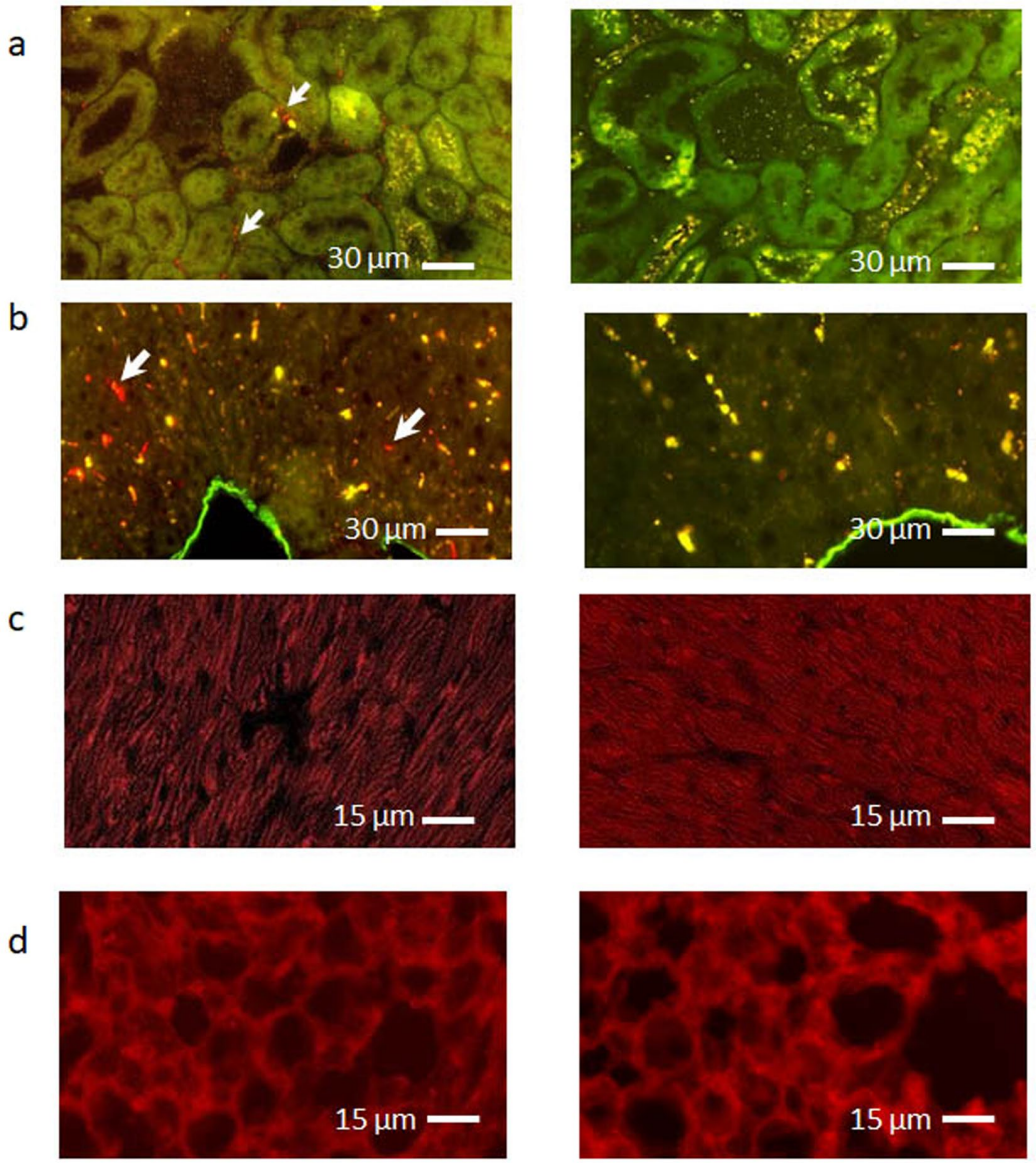
Exosomes localize to the kidney tubulointerstitium 30 min after injection. Photomicrographs on the left depict tissues 30 min after injection of PKH26-labeled exosomes (red), in mice with bilateral kidney ischemia. Images on the right are from tissues in mice with bilateral kidney ischemia, without injection of PKH-labeled exosomes (control). (**a**) Kidney: Exosomes are distributed mainly in the tubulointerstitial space (arrows). (**b**) Liver: Exosomes were also found within Kupffer cells (arrows). (**c**) Heart: Exosomes were not observed. (**d**) Lung: Exosomes were not observed. Considerable background autofluorescence (red) was observed in heart, liver and lung tissue, while green/yellow background autofluorescence was also seen in kidneys and liver. Images are representative of data from 6 mice.

To localize exosomes within kidney tissue, experiments were conducted in which exosomes were labeled with PKH26 prior to infusion. Figure 5 shows that 30 min after infusion into mice with kidney ischemia/reperfusion injury, PKH-labeled exosomes were detected mainly in the tubulointerstitial compartment of the kidney, surrounding peritubular capillaries. Intracellular localization was not detected. While PKH-labeled exosomes were observed within Kupffer cells of the liver at this time point, no exosomes were found in lung or heart.

### Role of CXCR4/SDF-1α interaction in exosome targeting

**Figure 6:**
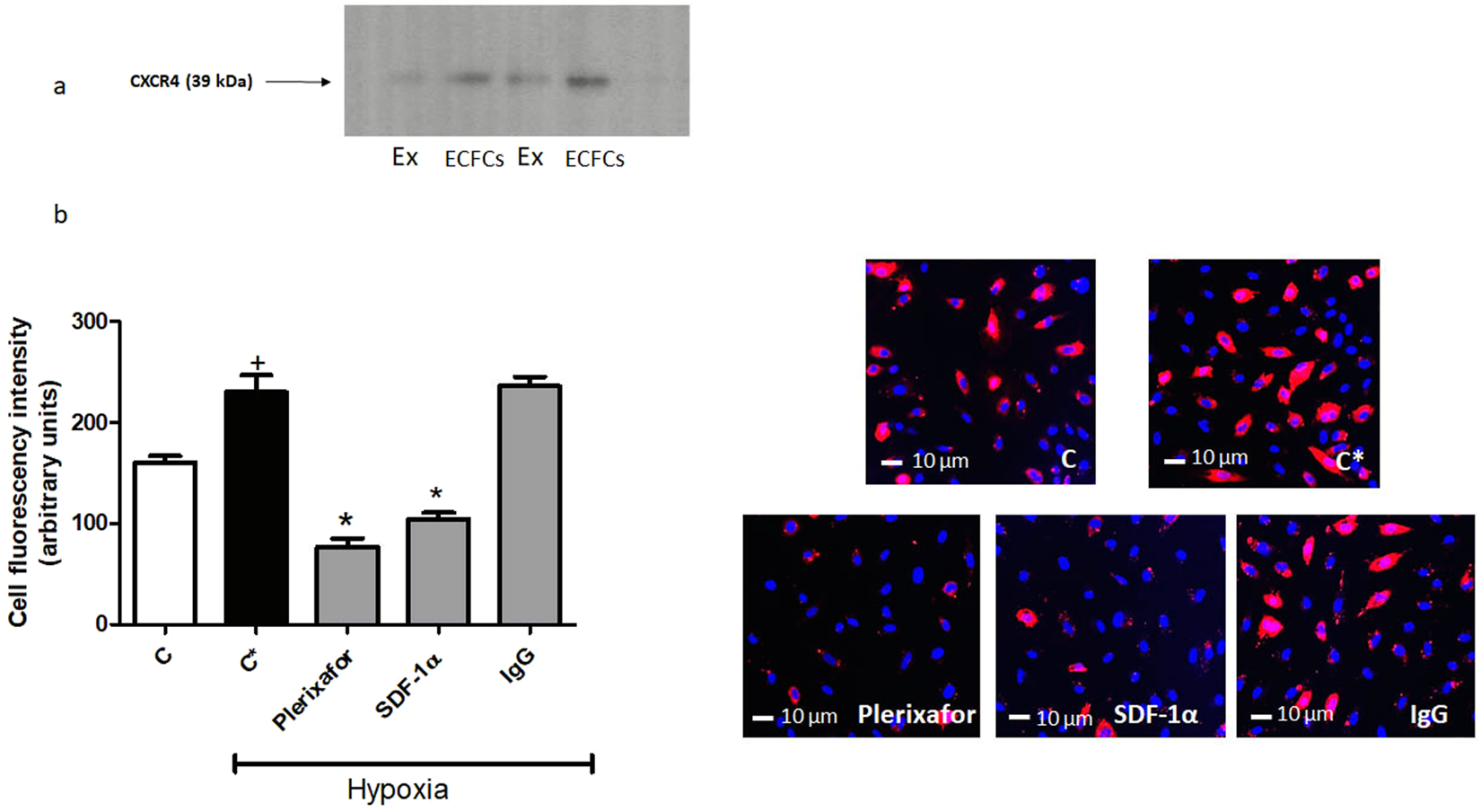
Internalization of endothelial colony forming cell (ECFC)-derived exosomes by hypoxic HUVECs is blocked by plerixafor and neutralizng antibody to SDF-1α. (**a**) Immunoblot shows expression of CXCR4 in ECFCs and their derived exosomes (EX). (**b**) Graph and representative images of HUVECs incubated for 6 hrs with 20 μg/ml of PKH26-labeled exosomes (red) in normoxia (**C**), hypoxia (C*), hypoxia with 100 μM plerixafor (Plerixafor), hypoxia with 10 μg/ml of neutralizing antibody to SDF-1α (SDF-1α) and Isotype control (IgG). HUVEC nuclear counterstaining was performed using Hoescht stain (blue). ^+^P < 0.001 vs C, *P < 0.001 vs C*, by one-way ANOVA, n = 4.

Biodistribution and real-time PCR studies in mouse tissues suggested that ECFC exosomes selectively target the kidneys after ischemia/reperfusion injury. *In vitro* studies were conducted to test the hypothesis that the CXCR4/SDF-1α axis might play a role in the targeting process, at the level of the endothelial cell. By immunoblot, exosomes expressed CXCR4, as did their parent ECFCs (Fig. 6a). In human umbilical vein endothelial cells (HUVECs) cultured under normoxic conditions, SDF-1α levels increased in the conditioned media in a time-dependent fashion, and exposure to hypoxia for 24 hrs further increased SDF-1α levels (Supp S2). Administration of PKH26-labeled exosomes to cultured HUVECs under normoxic conditions led to significant uptake of red fluorescence after 4 hrs, and exposure to hypoxia caused a further significant increase in exosome uptake (Fig. 6b). Incubation of hypoxic HUVECs with a neutralizing antibody to SDF-1α completely inhibited exosome uptake, while a control isotype antibody had no effect. In normoxic HUVECs, the CXCR4 inhibitor plerixafor significantly reduced the uptake of PKH26-labeled exosomes, in a dose-dependent fashion (Supp S3). Similarly, in hypoxic HUVECs, plerixafor (10^−4^ M) completely blocked exosome uptake (Fig. 6b).

### CXCR4/SDF-1α interaction mediates the exosomal transfer of miR-486-5p into cultured endothelial cells

**Figure 7:**
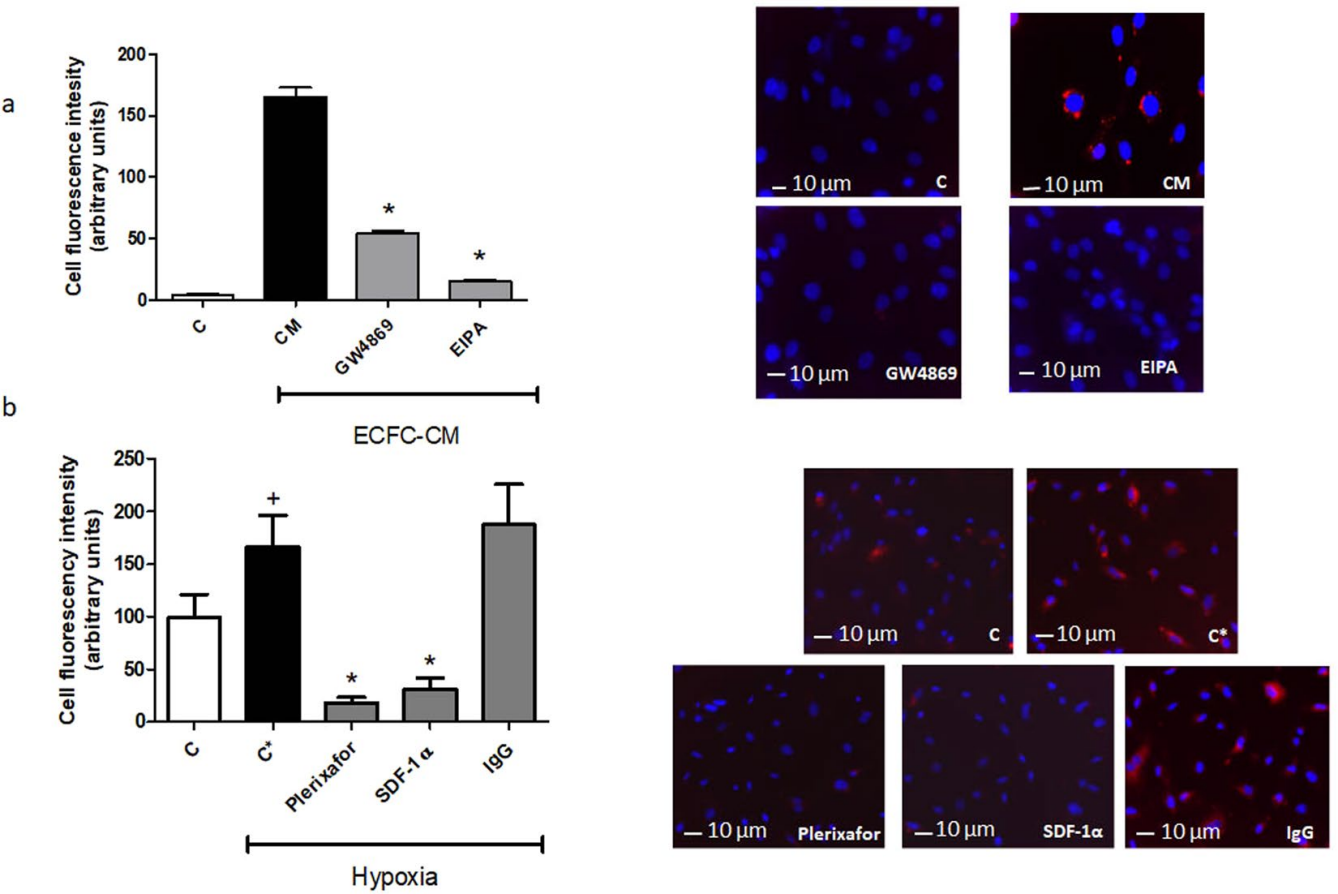
Uptake of exosomal Cy3-labeled pre-miR-486-5p by hypoxic HUVECs is prevented by plerixafor and neutralizing antibody to SDF-1α. (**a**) Graph and representative images of HUVECs after 16 hrs of incubation with conditioned medium from ECFCs (ECFC-CM) previously transfected with Cy3-pre-miR-486-5p (red). (C) control cells, (CM) HUVECs after incubation with conditioned medium from Cy3-pre-miR-486-5p transfected ECFCs, (GW4869) HUVECs incubated with conditioned medium from Cy3-pre-miR-486-5p transfected ECFCs, first treated with 10 μM GW4869 (inhibitor of exosome release), and (EIPA) HUVECs after incubation with conditioned medium from Cy3-pre-miR-486-5p transfected ECFCs, containing 10 μM EIPA (inhibitor of exosome uptake). *P < 0.001 vs CM, by one-way ANOVA, n = 4. (**b**) Graph and representative images from HUVECs after incubation with conditioned medium from Cy3-pre-miR-486-5p transfected ECFCs in normoxia (C), hypoxia (C*), hypoxia with 100 μM of plerixafor (Plerixafor), hypoxia with 10 μg/ml of neutralizing antibody to SDF-1α (SDF-1α) and 10 μg/ml Isotype control antibody (IgG). Red fluorescence is from Cy3-pre-miR-486-5p. HUVEC nuclear counterstaining was performed using Hoescht stain (blue). ^+^P < 0.05 vs C, *P < 0.01 vs C*, by one-way ANOVA, n = 4.

In previous co-culture experiments, we demonstrated the transfer of miR-486-5p from ECFCs to cultured HUVECs by qPCR^14^. Experiments were conducted to determine if the CXCR4/SDF-1α interaction mediates transfer of exosomal miR-486-5p into endothelial cells. ECFCs were transfected with Cy3-labeled pre-miR-486-5p, and after 24 hrs, the conditioned medium from these cells was administered to cultured HUVECs, under normoxic or hypoxic conditions. In normoxic HUVECs incubated with ECFC conditioned medium, Cy3 fluorescence was detected within the cytoplasm, an effect that was blocked by preincubation of ECFCs with the inhibitor of exosome release GW4869, or by incubation of HUVECs with the macropinocytosis inhibitor 5-(*N*-ethyl-*N*-iso-propyl)amiloride (EIPA), consistent with our previous real-time PCR experiments^14^ (Fig. 7a). Hypoxia caused a significant increase in uptake of Cy3 fluorescence in HUVECs, and incubation with either a neutralizing antibody to SDF-1α or plerixafor significantly inhibited the uptake of Cy3-labeled miR-486-5p (Fig. 7b).

### Effect of exosomal CXCR4 on ischemic kidney injury

**Figure 8:**
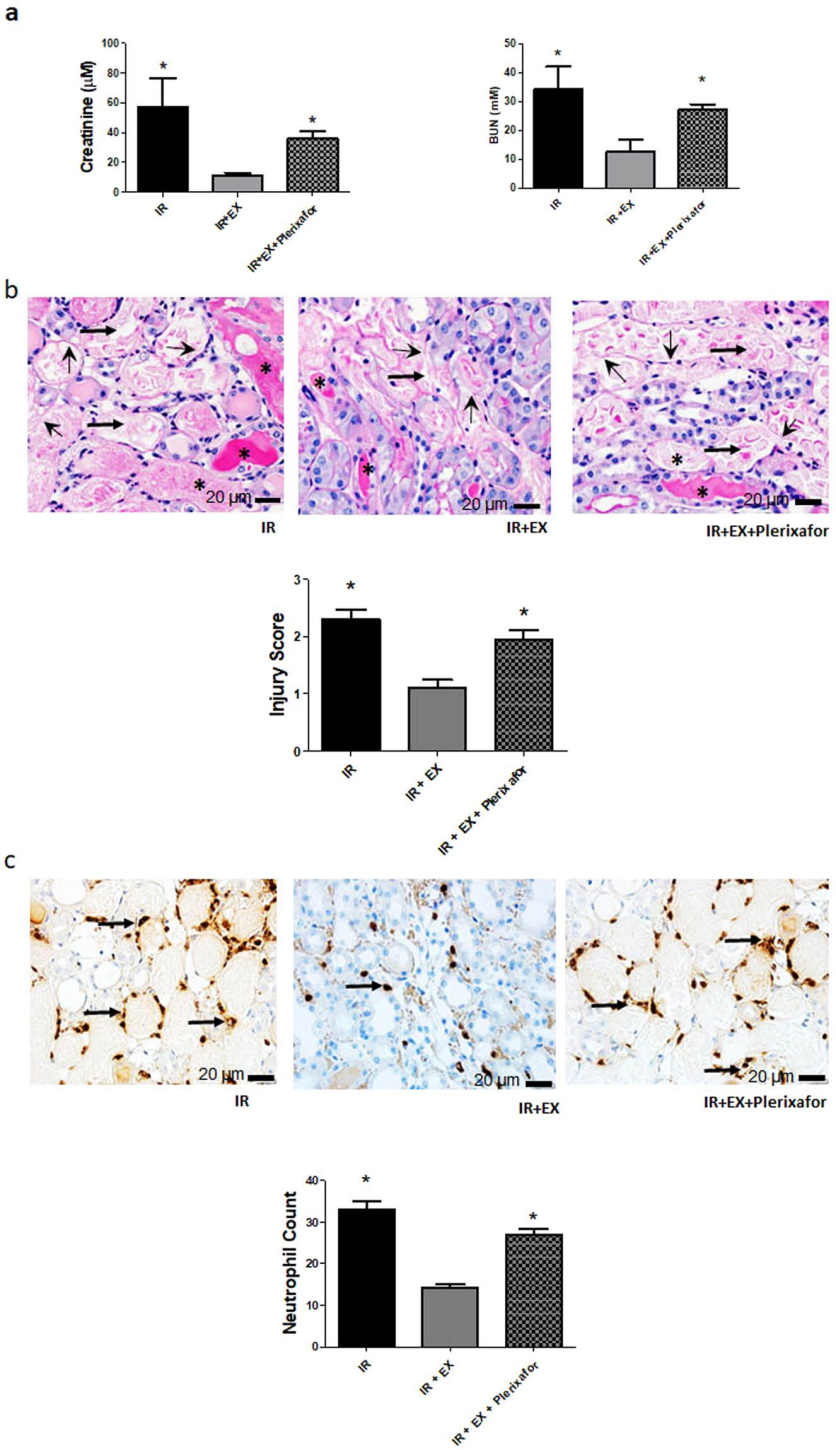
Effect of exosomal CXCR4 on kidney ischemic injury. Graphs depict data obtained 24 hrs after reperfusion in mice subjected to ischemic kidney injury (IR), with or without i.v. administration of endothelial colony forming cell exosomes (IR+EX), or exosomes pre-incubated with the CXCR4 antagonist plerixafor (IR+EX+Plerixafor). (**a**) Plasma Cr and Blood Urea Nitrogen (BUN) levels 24 hrs after reperfusion. *P < 0.05 vs IR+EX. (**b**) Kidney histologic injury scores for mice with kidney ischemia, 24 hrs after reperfusion, with or without exosomes. *P < 0.001 vs IR+EX; Representative photomicrographs are shown above graph. Astericks (*) depict tubular cast formation, arrows depict hallmark tubular dilatation, and arrowheads depict nuclear loss. (**c**) Quantitative assessment of neutrophil infiltration in kidneys (number of neutrophils per field), for mice with kidney ischemia, 24 hrs after reperfusion, with or without exosomes. *P < 0.001 vs IR+EX. Representative photomicrographs are shown above graph. Arrows depict peritubular neutrophil infiltrate. Data are from 4 mice in each group, except for IR alone, (n = 6 mice).

Further experiments were performed to determine the effect of exosomal CXCR4 on kidney ischemic injury *in vivo*. Mice were subjected to bilateral renal artery occlusion for 30 min, followed by tail vein infusion of vehicle, ECFC exosomes (20 µg), or ECFC exosomes (20 µg) that had been previously incubated with plerixafor (10^−4^ M) for 3 hrs at 37 °C. As shown in Fig. 8, ischemia-reperfusion increased plasma Cr and blood urea nitrogen (BUN) after 24 hrs, and was associated with significant kidney histologic injury and neutrophil infiltration, effects that were prevented in mice treated with ECFC exosomes. By contrast, infusion of mice with ECFC exosomes pre-incubated with plerixafor had no protective effect on the extent of kidney injury.

## Discussion

The major finding of the current study is that intravenously delivered ECFC-derived exosomes selectively target the kidneys after ischemic injury, leading to increased levels of miR-486-5p within kidney glomeruli, proximal tubule and endothelial cells. The data in cultured HUVECs suggest that selective targeting involves interaction of exosomal CXCR4 with SDF-1α elaborated by endothelial cells, a process that is also necessary for exosomal transfer of miR-486-5p. In this regard, ECFC exosomes are enriched in miR-486-5p, which targets PTEN and contributes to inhibition of apoptosis and reduced ischemic injury^14^. Finally, we show that pre-incubation of exosomes with an inhibitor of CXCR4, plerixafor, results in loss of protection against kidney ischemic injury *in vivo*.

The mobilization and selective homing of endogenous EPCs from bone marrow to sites of tissue injury has been implicated in the formation of new blood vessels. This process of neovascularization involves chemoattractant signals released by injured cells, including SDF-1α, angiopoietins, and stem cell factor^15,16^, and interaction with cell surface proteins expressed on EPCs, including integrins and CXCR4^10,17,18^. ECFCs are late outgrowth EPCs that have profound protective properties when administered exogenously in experimental models of ischemic kidney injury^5,6^. However, recent studies suggest that cell engraftment does not occur following ECFC administration, and release of exosomes and other soluble factors may mediate the beneficial effects of cell therapy^6,14^.

Using optical imaging with the near infrared dye DiR, we demonstrated that infused labeled exosomes selectively localized to the kidneys 30 min and 4 hrs after ischemia/reperfusion. Free-hand quantification of fluorescence intensity in regions-of-interest showed accumulation of fluorescence in the abdomen by posterior view, and *ex vivo* analysis of dissected organs confirmed uptake into the kidneys. By contrast, labeled exosomes did not preferentially target the heart, liver, lungs or spleen by optical imaging, and mice without AKI did not exhibit kidney-specific biodistribution. In agreement with these data, in a mouse model of glycerol-induced AKI Grange *et al*. used optical imaging to show that extracellular vesicles derived from mesenchymal stem cells accumulated specifically within the kidneys 5 hrs and 24 hrs after intravenous injection, with variable uptake into liver and spleen^19^. While we did not observe kidney-specific localization of labeled exosomes 24 hrs after ischemia/reperfusion injury, kidney levels of miR-486-5p remained persistently elevated 24 hrs after exosome infusion, compared to mice with AKI without exosome infusion. These data suggest that cellular uptake and degradation of exosomes was completed by 24 hrs, preceded by cellular transfer of miR-486-5p.

In the present studies, exosomes labeled with PKH26 were localized primarily to the kidney interstitium shortly after infusion in mice with AKI, with variable fluorescence found within glomeruli. Endothelial cell-specific localization was not found in kidneys, nor did we observe fluorescent staining within tubular cells. While PKH26-labeled exosomes were detected in hepatic Kupffer cells, no fluorescence was found in heart or lung tissue. In rats with ischemia/reperfusion AKI, Cantaluppi *et al*. localized PKH26-labeled microvesicles derived from human EPCs to endothelial cells of large vessels and peritubular capillaries, tubular epithelial cells, glomeruli, and liver, 2 or 6 hrs after intravenous injection, by confocal microscopy^13^. In mice with ischemic kidney injury, intravenously administered microparticles (submicron diameter) from kidney-derived mesenchymal stem cells were localized to peritubular capillaries 3 days after injection^20^. The origin and characteristics of the extracellular vesicles, time of sacrifice, and species differences may account for the varying exosome localization in these studies. Nonetheless, our study suggests that ECFC exosomes traversed the endothelial cell barrier as early as 30 min after infusion, perhaps related to increased vascular permeability in the ischemic kidney.

Exosomes contain miRs, mRNA, proteins and other cargo and act as paracrine mediators of tissue repair. In this regard, depletion of pro-angiogenic miR-126 or miR-296 from EPC-derived microvesicles has been shown to abolish the protective effect of the vesicles in rats with ischemic kidney injury, suggesting that the miR cargo transfers to resident cells within the kidneys and induces a regenerative program^13^. In mice with ischemia/reperfusion injury, we showed that infusion of ECFC exosomes was renoprotective, associated with increased kidney levels of miR-486-5p, inhibition of PTEN expression, and enhanced phosphorylation of Akt^14^. These effects were blocked with infusion of exosomes derived from ECFCs that were first transfected with antagomiR to miR-486-5p. Here, we show early selective increases in miR-486-5p within kidney cortex after ischemic injury, and specific increases within proximal tubular cells, glomeruli, and kidney endothelial cells. These data provide compelling evidence for exosomal transfer of miR-486-5p to these cells. Furthermore, while uptake of exosomes into cells may occur via a number of endocytic mechanisms, including clathrin-mediated endocytosis and lipid raft-mediated internalization^21^, uptake of Cy3-labeled miR-486-5p into HUVECs was inhibited by EIPA, suggesting a major role for the macropinocytosis pathway.

SDF-1α is a critical chemokine involved in vascular repair, and is secreted by endothelial cells exposed to hypoxic injury^11^. The interaction and signaling between up-regulated SDF-1α and its receptor CXCR4 has been proposed to play a central role in the homing of bone marrow-derived EPCs to sites of tissue injury. In mice with ischemia/reperfusion kidney injury, kidney expression of SDF-1α increases, and mediates homing and migration of CXCR4 expressing CD34-positive cells from the bone marrow to the injured tissue^22^. However, the mechanisms of selective tissue targeting by exogenously administered exosomes remain poorly characterized. Exosomes and other extracellular vesicles are thought to interact with the plasma membrane of target cells by rolling, followed by binding of specific vesicle membrane proteins with their cell receptors, fusion to the target plasma membrane, and release of cargo molecules into the cytoplasm^23^. Binding and fusion to plasma membrane also occurs in endocytic processes^23^. Kang *et al*. overexpressed CXCR4 in mesenchymal stem cells and demonstrated that exosomes derived from these cells were enriched in CXCR4 and had increased capacity to reduce left ventricular remodelling and restore function after myocardial infarction in rats^24^. In the present studies, CXCR4 was expressed in both ECFCs and exosomes, and SDF-1α secretion was enhanced by exposure of HUVECs to hypoxia. Importantly, exosome uptake into HUVECs exposed to hypoxia was increased compared to normoxic cells, and was significantly inhibited by incubation with the CXCR4 inhibitor plerixafor, or by a neutralizing antibody to SDF-1α. The CXCR4/SDF-1α interaction is necessary for transfer of miR-486-5p into HUVECs, since both plerixafor and neutralizing antibody to SDF-1α blocked uptake of Cy3-labeled miR. The importance of CXCR4 is further supported by our *in vivo* data, which revealed that blockade of exosomal CXCR4 prior to intravenous infusion completely eliminated the protective effects of exosomes on kidney ischemic injury, determined by plasma Cr and BUN, as well as histologic measures and extent of neutrophil infiltration. Taken together, these data suggest a role for exosomal CXCR4 interaction with SDF-1α in the targeting of exosomes to kidney endothelium after ischemia. The mechanisms in which this interaction enhance exosome mobilization are unclear, but could involve response to a gradient established by SDF-1α, and/or direct interaction between CXCR4 and SDF-1α at the plasma membrane, promoting exosome uptake. Nonetheless, other pathways may also contribute to selective targeting, involving exosome integrin expression, or target cell surface expression of adhesion molecules such as ICAM-1, as has been described for B cell-derived exosomes^25^.

MicroRNAs have been implicated in the development and progression of experimental and human acute and chronic kidney diseases^26^. Using laser-capture microdissection and deep sequencing of small RNA, miR-486-5p levels were found to be significantly down-regulated within glomeruli and proximal tubules in kidney biopsies from patients with diabetic nephropathy and IgA nephropathy, and within proximal tubules from patients with focal and segmental glomerulosclerosis^27^. While the consequences of diminished miR-486-5p expression in various kidney compartments to disease progression remain unclear, targeting of PTEN by miR-486-5p is associated with improved endothelial cell survival^14^. Dellett *et al*. have also demonstrated that ECFC-derived extracellular vesicles contain high levels of miR-486-5p, and stimulate angiogenesis in a mouse model of oxygen-induced retinopathy^28^. Thus, strategies to enhance delivery and targeting of ECFC exosomes to injured tissues could have broader therapeutic implications beyond effects in ischemic kidney injury.

## Methods

### Cell culture

ECFCs were cultured (passages 3-5) from human umbilical cord blood units following informed written consent, with approval from the Canadian Blood Services Research Ethics Board and characterized as described^5,14^. HUVECs were obtained from the American Type Culture Collection (Manassas, VA, USA). ECFCs and HUVECs were cultured in Endothelial Cell Growth Medium EndoMax (Wisent Inc., St-Bruno, QC, Canada), or Endothelial basal cell growth Medium 2 with SingleQuots supplements (Lonza, Basel, Switzerland), supplemented with 10% fetal bovine serum. All experiments were performed in accordance with the policies and regulations of the Ottawa Hospital Research Institute and the University of Ottawa.

### Exosome isolation and characterization

Exosomes were isolated from ECFC-conditioned medium by successive centrifugations, after 24 hrs of culture in the presence of vesicle-depleted FBS (Wisent Inc.), as we have described^5,14^. Sizing of exosomes was performed by nanoparticle tracking analysis using the Nanosight LM10 instrument (NanoSight Ltd., Amesbury, UK)^5,14^, and ZetaView (Particle Metrix, Meerbusch, Germany).

### Immunoblots

Exosome lysates were run on 10% PAGE gels and membranes were incubated with antibodies against TSG101 (1:1000, Abcam Inc., Toronto, ON, Canada), CD81 (1:1000, Abcam Inc.), and monoclonal antibodies against CXCR4 (1:1000, Abcam Inc.). Washed membranes were incubated with horseradish peroxidase-conjugated secondary antibodies (1:2000, Jackson Immunoresearch, West Grove, PA, USA), and chemiluminescence detected using a Konica Minolta SRX 101 A Tabletop Medical Film Processor (Konica Minolta Medical Imaging USA Inc., Wayne, NJ, USA). Data were analyzed using ImageJ software (NIH, Bethesda, MD, USA), and were corrected for loading controls (GAPDH, 1:1000, Millipore, Toronto, ON, Canada).

### Kidney ischemia/reperfusion model

Bilateral kidney ischemia/reperfusion was performed on 7–10-week old male FVB mice (Charles River, St. Constant, QC, Canada) with renal vascular clamp for 30 min, essentially as described^5,14^. For experiments involving measurements of miR-486-5p levels by real-time PCR in tissues, mice received either vehicle (100 µL PBS) or ECFC-derived exosomes (20 µg in 100 µL PBS) via tail vein injection. Some experiments involved infusion of exosomes that had been previously incubated for 3 hrs at 37 °C with the CXCR4 inhibitor plerixafor (10^−4^ M) (AMD3100, EMD Millipore Corp., Darmstadt, Germany). In some experiments conducted 24 hrs after reperfusion, exosomes were infused via jugular vein infusion at the time of reperfusion. Sham mice were subjected to surgery without renal vascular clamping. Protocols were approved by the Animal Ethics Committee at the University of Ottawa and performed according to the recommendations of the Canadian Council for Animal Care.

### Plasma Biochemistry

Plasma creatinine and BUN levels were measured by IDEXX Laboratories (Markham, ON, Canada), as we have previously done^5,14^.

### Histology

Twenty four hours after reperfusion, mice were sacrificed and kidneys were fixed in 4% formalin, dehydrated, embedded in paraffin, and stained as described^5^. Histologic injury was performed semi-quantitatively with scoring on a scale of 0–4 ^5^. Neutrophil infiltration was assessed on kidney sections by quantitation of myeloperoxidase staining (polyclonal rabbit myeloperoxidase antibody, 1:200, Neomarker, Fremont, CA)^14^. All assessments were performed with the pathologist (AG) blinded to the experimental groups. Images were acquired at room temperature on a Zeiss Imager A1 with a Zeiss AxioCam HRc using Axiovision version 1.6 (Carl Zeiss AG, Oberkochen, Germany).

### Optical imaging

The biodistribution of infused ECFC-derived exosomes was studied in anesthetized mice and organs extracted *ex vivo*, using the IVIS Spectrum *in vivo* imaging system (PerkinElmer Inc., Waltham, MA, USA). Exosomes were first labeled with DiR, using a method involving serial centrifugations of 100,000 x g, as per the manufacturer’s recommendation (Invitrogen, Carlsbad, CA, USA). The labeled exosomes (20 µg/mouse) were infused into anesthetized mice via tail vein at various time points after ischemia-reperfusion. Some mice received infusions of DiR alone (1.7 µL/100 µL PBS). Fluorescence imaging was conducted in the IVIS system at 710 nm of excitation and 760 nm of emission. The intensity of the region of interest (ROI), drawn by freehand, was plotted in units of the maximum number of photons per second per centimeter square per steridian (p/s/cm^2^/sr). In some mice, organs were dissected *ex vivo* and subjected to IVIS imaging for measurement of fluorescence. In all studies, background fluorescence was subtracted from values obtained from mice that had not been infused with DiR or DiR-labeled exosomes. Data were collected using the Living Image Software (version 4.3.1) from IVIS Spectrum.

### miRNA isolation and real-time PCR

Total RNA inclusive of the small RNA fraction was extracted from organs and kidney regions/segments using the miRNeasy Mini Kit (Qiagen Inc., Toronto, ON, Canada). The TaqMan®MicroRNA Reverse Transcription Kit (Life Technologies Inc., Carlsbad, CA, USA) was used for cDNA preparation. Real-time PCR reactions were performed in an Applied Biosystems 7000 sequence detection real-time PCR system (Foster City, CA, USA). Mamm-U6 RNA was used to normalize differences in RNA levels of each sample. The relative amount of miRNA to U6 RNA was expressed using the 2−ΔΔCt method^29^.

### Isolation of kidney cortices, medullae, proximal tubule segments and glomeruli

In some experiments, kidneys were removed and decapsulated 30 min after reperfusion, cortices and medullae were surgically separated, and miRNA extracted from both sections. Additional dissection of glomeruli and proximal tubules was performed, as described^30^. Briefly, kidney cortices from mice were dissected, and gently minced in a glass petri dish on ice. The tissue was then suspended in a solution containing (in mM) 105 NaCl, 24 NaCO_3_, 5 KCl, 1.5 CaCl_2_, 1.0 MgSO_4_, 2.0 NaH_2_PO_4_, 5.0 glucose, 1.0 alanine, and 10.0 HEPES, pH 7.4, as well as 0.1% collagenase (type IV, Sigma, Oakville, ON, Canada). The suspension was gassed with 95% O_2_-5% CO_2_ for 30 min in a 37 °C water bath. After digestion, the cortical suspension was strained through a 250-µm brass sieve (mesh no. 60, Newark Wire Cloth, ESBE Scientific, Markham, ON, Canada) and centrifuged for 1 min at 1,000 × g. The pellet was resuspended in the same solution without collagenase and centrifuged for 1 min, repeated once. After the tissue was strained through a 106-µm brass sieve (mesh no. 150), the proximal tubules were microdissected from the tissue on top of the sieve, and glomeruli were microdissected from the tissue collected on a 75-µm brass sieve (mesh no. 200) immediately below the 106-µm sieve. The purity of the tubular and glomerular preparations (>99%) was determined by light microscopy.

### Isolation of kidney endothelial cells

Whole kidneys were dissociated into a single cell suspension by enzymatic digestion using a Gentlemacs dissociator device (Miltenyi Biotec GmbH,  Bergisch Gladbach, Germany). Endothelial cells were sorted using CD31-labeled magnetic beads (Miltenyi Biotec GmbH), according to the manufacturer’s instructions. The sorted cells were subjected to miRNA extraction and real-time PCR as described above. An aliquot of cells was cultured to demonstrate immunofluorescent staining for the endothelial cell surface marker CD31.

### Localization of PKH26 exosomes within tissues

To localize exosomes in tissues, exosomes were first labeled for 5 min at room temperature with the red fluorescent dye PKH26 (Sigma), subjected to ultracentrifugation, and then resuspended in PBS. PKH26-labelled exosomes (20 µg) were administered to mice, with or without kidney ischemia/reperfusion, by tail vein injection. After 30 min, the mice were sacrificed, and the kidneys, liver, heart, lung, and spleen were harvested. Tissues were embedded in OCT and stored in liquid nitrogen. Cryostat sections (20 µm thickness) were prepared using a freezing microtome (Leica CM3050 S; Leica Biosystems, Wetzlar, Germany), fixed in 3% paraformaldehyde for 10 min, washed in PBS three times for 10 min each, and mounted in VectaShield (Vector Labs, Burlingame, CA, USA). The sections were examined in a blinded fashion by a pathologist (AG), using a fluorescent microscope (Zeiss AxioObserver Z1, Zeiss, Oberkochen, Germany).

### Cellular hypoxia

In some experiments, HUVECs were rendered hypoxic for 24 hrs (0.5% O_2_) in a humidified hypoxic chamber (H35 Hypoxystation HypO2xygen, Don Whitley Scientific, Shipley, West Yorkshire, UK).

### Exosome uptake into HUVECs

PKH26-labeled exosomes were administered to cultured HUVECs (in normoxic or hypoxic conditions), followed by measurement of cytoplasmic fluorescence after 4–6 hrs. In some experiments HUVECs were first treated with the CXCR4 inhibitor plerixafor, a neutralizing antibody against SDF-1α (10 µg/ml) (R&D Systems, Minneapolis, MN, USA) or the same concentration of a control isotype antibody (R&D Systems).

### Cell transfection

ECFCs were transfected with pre-miR-486-5p (Life Technologies, Inc.) labeled with Label IT® siRNA Tracker Cy®3 (Mirus Bio, Madison, WI, USA), using Lipofectamine RNAiMAX Reagent (Invitrogen). After 24 hrs, the conditioned medium was removed and applied to HUVECs cultured on 6-well dishes for 16 hrs. In these experiments, ECFCs were first treated with or without the inhibitor of exosome release GW4869^31^ (10 µM, Cayman Chemical, Ann Arbor, MI, USA), and HUVECs were incubated with or without the macropinocytosis inhibitor EIPA (10 µM, Sigma)^32^.

### SDF-1α assay

In cultured HUVECs, secreted SDF-1α in the conditioned media was measured using an ELISA kit (RayBiotech, Norcross, GA, USA), following the manufacturer’s instructions. The concentration of SDF-1α was calculated by measuring the absorbance at 450 nm, using a microplate reader.

### Statistical analysis

Results are expressed as mean ± SEM and were analyzed using Student t-test, or one-way ANOVA with Bonferroni’s post-test or Kruskal-Wallis test as appropriate. Statistical analyses were performed with GraphPad Prism 5.0 (GraphPad Software, Inc., San Diego, CA, USA). P < 0.05 was considered significant.

## Electronic supplementary material

Supplementary Figures

Supplementary Figure 1 (S1)
