# Supplementary material for: Receptor-Ligand Interaction Mediates Targeting of Endothelial Colony Forming Cell-derived Exosomes to the Kidney after Ischemic Injury
Source: Sci Rep. 2018 Nov 5;8:16320. doi: 10.1038/s41598-018-34557-7 (PMC6218514; doi:10.1038/s41598-018-34557-7)
Supplement: Supplementary file 1 — Supplementary Figures [file 41598_2018_34557_MOESM1_ESM.pdf]

Receptor-Ligand Interaction Mediates Targeting of Endothelial Colony Forming Cell-derived Exosomes to the Kidney after Ischemic Injury

Jose L. Viñas[1], Matthew Spence[1], Alex Gutsol[1], William Knoll[1], Dylan Burger[1], Joseph Zimpelmann[1], David S. Allan[2] and Kevin D. Burns*[1]

From the Division of Nephrology, Dept. of Medicine, Kidney Research Centre, Ottawa Hospital Research Institute, University of Ottawa[1], and the Division of Hematology, Dept. of Medicine, Ottawa Hospital Research Institute, University of Ottawa[2], Ottawa, Ontario, Canada

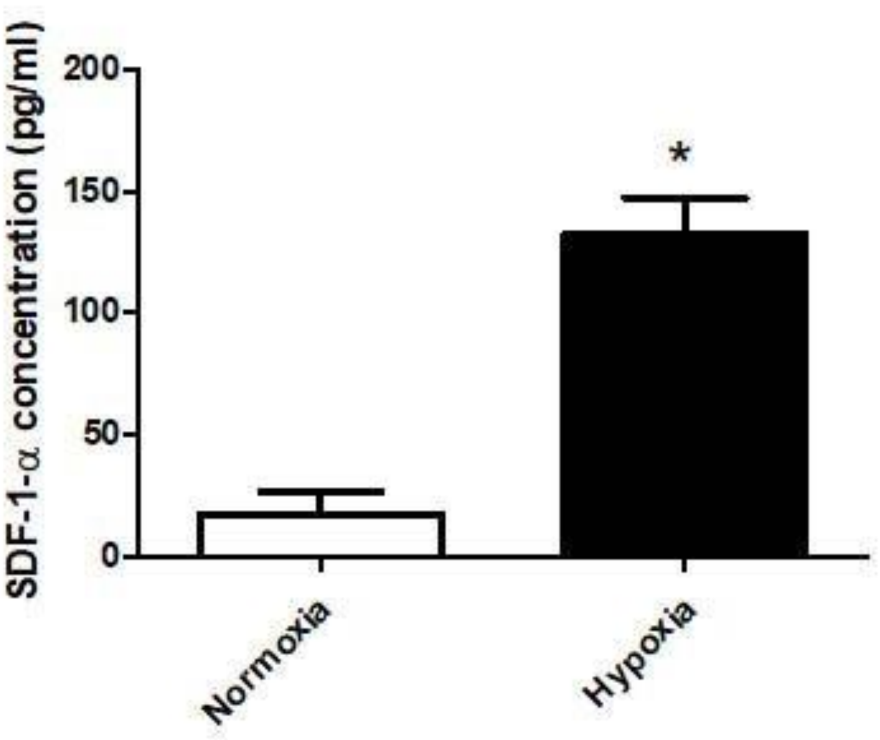

<u>Supplementary Figure 2 (S2):</u> **Hypoxia increases secretion of stromal cell-derived factor (SDF)-1α in cultured endothelial cells.**

Graph depicts SDF-1α levels in cell culture supernatants from normoxic HUVECs (Normoxia) and HUVECs subjected to 24 hrs of hypoxia (Hypoxia). *P<0.001 vs Normoxia, by Student t-test, n=5.

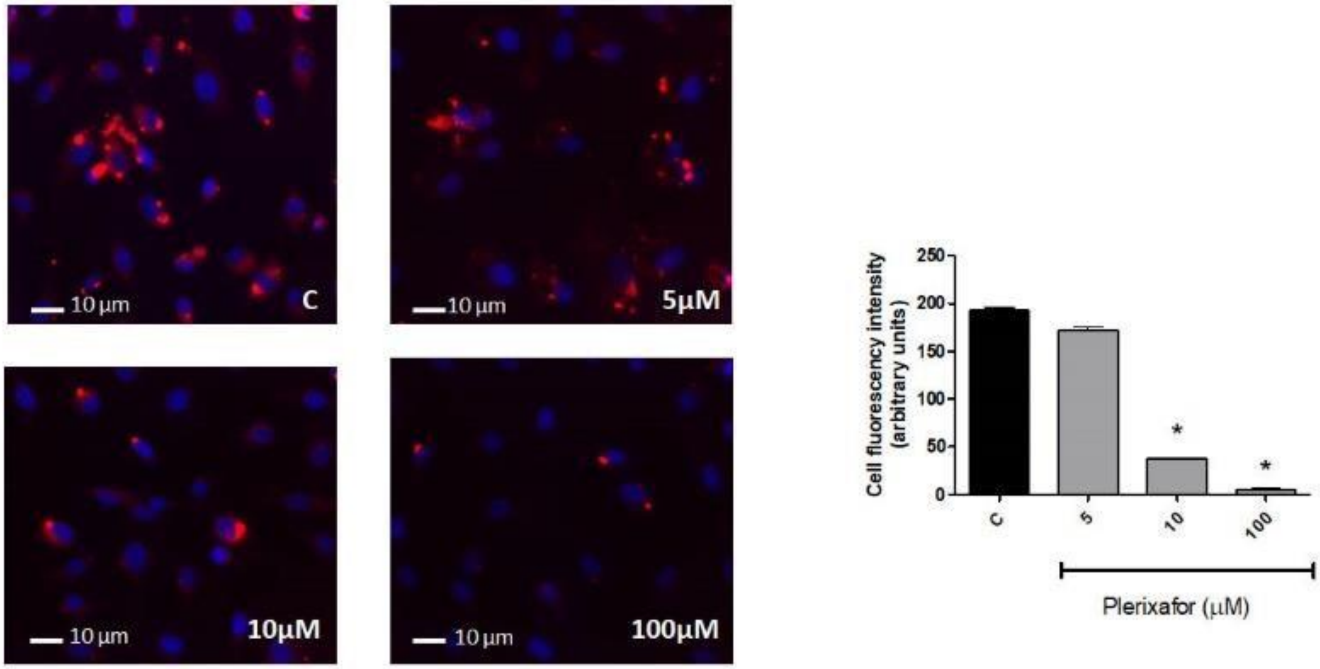

<u>Supplementary Figure 3 (S3):</u> **Dose-dependent inhibition of exosome uptake into endothelial cells by the CXCR4 antagonist plerixafor.**

Cultured HUVECs were incubated with 20 µg/ml of PKH26-labeled exosomes in the presence or absence of plerixafor. (C) Untreated HUVECs after incubation with PKH26 exosomes for 6 hrs, (5) + 5 µM plerixafor, (10) + 10 µM plerixafor and (100) + 100 µM plerixafor. *P<0.001 vs C, by Student t-test, n=4.

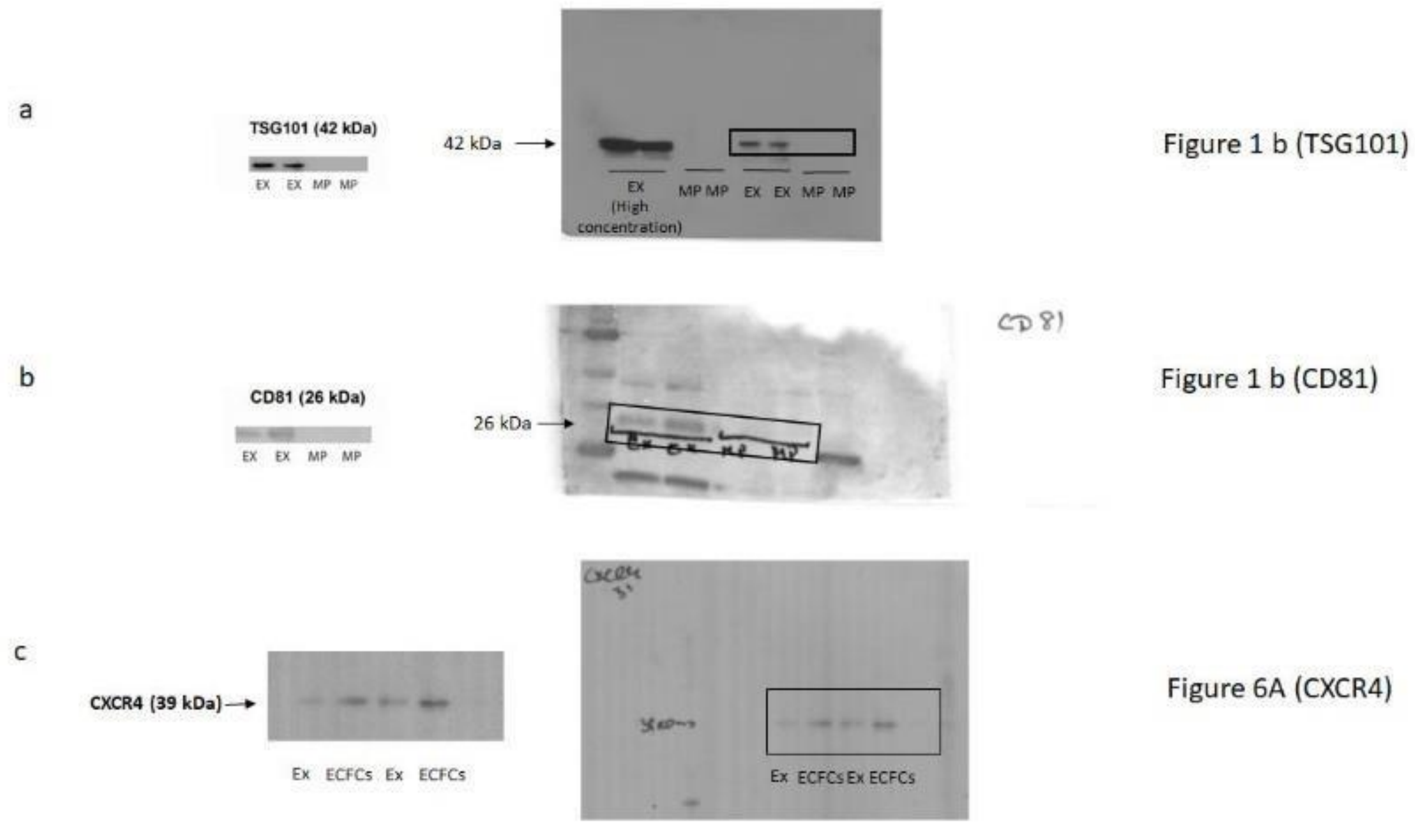

Supplementary Figure 4 (S4): Images of entire immunoblots depicting TSG101 (a), CD81 (b) in exosome (EX) and microparticle (MP) preparations (see Figure 1), and CXCR4 in ECFCs and their exosomes (Fig. 4c).
